# Human pancreatic ductal organoids with controlled polarity provide a novel ex vivo tool to study epithelial cell physiology

**DOI:** 10.1007/s00018-023-04836-2

**Published:** 2023-06-28

**Authors:** Árpád Varga, Tamara Madácsy, Marietta Görög, Aletta Kiss, Petra Susánszki, Viktória Szabó, Boldizsár Jójárt, Krisztina Dudás, Gyula Farkas, Edit Szederkényi, György Lázár, Attila Farkas, Ferhan Ayaydin, Petra Pallagi, József Maléth

**Affiliations:** 1 Department of Medicine, University of Szeged, Szeged, Hungary; 2 ELRN-USZ Momentum Epithelial Cell Signaling and Secretion Research Group, Department of Medicine, University of Szeged, Szeged, 6720 Hungary; 3 HCEMM-USZ Molecular Gastroenterology Research Group, University of Szeged, Szeged, Hungary; 4 Department of Surgery, University of Szeged, Szeged, Hungary; 5 HCEMM-USZ Functional Cell Biology and Immunology Advanced Core Facility, University of Szeged, Szeged, Hungary

**Keywords:** Apical-out, 3D culture, Ductal cell, Organoid, CFTR

## Abstract

Epithelial ion and fluid secretion determine the physiological functions of a broad range of organs, such as the lung, liver, or pancreas. The molecular mechanism of pancreatic ion secretion is challenging to investigate due to the limited access to functional human ductal epithelia. Patient-derived organoids may overcome these limitations, however direct accessibility of the apical membrane is not solved. In addition, due to the vectorial transport of ions and fluid the intraluminal pressure in the organoids is elevated, which may hinder the study of physiological processes. To overcome these, we developed an advanced culturing method for human pancreatic organoids based on the removal of the extracellular matrix that induced an apical-to-basal polarity switch also leading to reversed localization of proteins with polarized expression. The cells in the apical-out organoids had a cuboidal shape, whereas their resting intracellular Ca^2+^ concentration was more consistent compared to the cells in the apical-in organoids. Using this advanced model, we demonstrated the expression and function of two novel ion channels, the Ca^2+^ activated Cl^−^ channel Anoctamin 1 (ANO1) and the epithelial Na^+^ channel (ENaC), which were not considered in ductal cells yet. Finally, we showed that the available functional assays, such as forskolin-induced swelling, or intracellular Cl^−^ measurement have improved dynamic range when performed with apical-out organoids. Taken together our data suggest that polarity-switched human pancreatic ductal organoids are suitable models to expand our toolset in basic and translational research.

**Graphical abstract:**

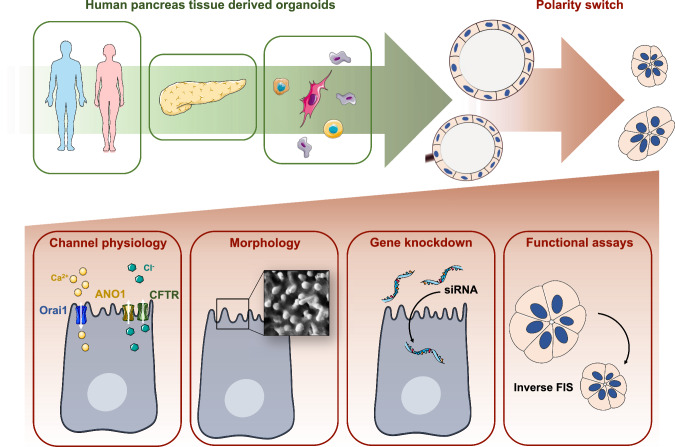

**Supplementary Information:**

The online version contains supplementary material available at 10.1007/s00018-023-04836-2.

## Introduction

Secretory epithelial cells act as a barrier between the lumen of different organs and the rest of the body, whereas they govern ion and fluid secretory processes to determine the amount and composition of the secreted juice. The pancreatic ductal epithelial cells secrete ~ 2 L HCO_3_^−^ rich alkaline fluid daily that is essential to maintain the physiological operation of the pancreas by flushing out bioactive molecules (such as digestive enzymes) from the ductal tree to the duodenum, preventing early proenzyme activation and thus tissue damage or failure [1–3]. On the other hand, impaired ductal secretion is a common feature in distinct pathophysiological conditions of the pancreas such as the genetic disorder cystic fibrosis (CF) and acute or chronic pancreatitis [4–8]. The vectorial ion and fluid secretion of the ductal cells are largely determined by the expression of ion channels and transporter proteins that display a strictly regulated apical-to-basal polarity [1, 9]. According to the currently accepted model of ion secretion, the pancreatic ductal cells express the cystic fibrosis transmembrane regulator (CFTR) Cl^−^ channel and SLC26A6 Cl^−^/HCO_3_^−^ exchanger on the apical membrane [1]. The functional interplay of these proteins and the physical interaction of the R domain of CFTR and STAS domain of SLC26A6 enable the pancreatic ductal cells to generate a remarkably high intraluminal HCO_3_^−^ concentration that is ~ 140 mM in humans [10]. However, currently the mechanism of human HCO_3_^−^ secretion is still debated and not completely understood.

One major bottleneck in the study of pancreatic secretory processes is the lack of human-relevant model systems that provide access to the apical membrane of ductal cells. Although, adherent cell lines and animal models are widely used and universal tools in both basic research and biomedical drug discovery they all have certain limitations [11]. Due to frequent species-specific differences, extrapolation of results from models or clinical translation of basic research findings remained a major unmet need. The recently developed patient-derived organoid cultures may provide a physiologically more relevant platform resolving previous difficulties [12–14]. Epithelial organoids are ex vivo three-dimensional (3D) cell cultures originated from Leucine-rich repeat-containing G-protein coupled receptor 5 positive (LGR5+) adult stem cells, which shows high self-renewal capacity in general [15, 16]. Organoids can be established from human tissue samples and can be maintained for several passages making it easier to conduct studies where the availability of primary cells is limited, such as in the case of the lung or pancreas [12, 17]. Our group previously demonstrated that the expression, localization and function of key proteins involved in ductal fluid and ion secretion (CFTR, Na^+^/H^+^ exchanger 1—NHE1 and electrogenic Na^+^/HCO_3_^−^ cotransporter 1—NBCe1) are equivalent in the pancreatic organoids and the primary isolated ductal fragments in mice [18]. Moreover, the HCO_3_^−^ secretion and CFTR activity are equivalent in the pancreatic organoids and the isolated ductal fragments. However, direct accessibility of the apical membrane which would be essential for basic physiological investigations and to develop drug screening assays is still difficult to implement. In addition, due to the vectorial ion and fluid transport into the lumen of the organoids, the increasing intraluminal pressure alone may be sufficient to negatively regulate epithelial secretory or barrier functions via Piezo1 mechanosensitive receptor [19]. One possible strategy to overcome this could be the manipulation of the cell polarity in organoids, which was successfully applied in intestinal and airway organoids by the removal of the extracellular matrix (ECM) to investigate host–pathogen interactions and infectious diseases [20–22]. However, it is currently unknown how the polarity switch may alter the epithelial cell functions.

Therefore, we aimed to establish an advanced culturing method for human pancreas-derived organoids based on ECM-removal-induced apical-to-basal polarity switching. Our results demonstrated that the cell and nuclei diameter in the apical-out organoids decreased indicating the elimination of the intraluminal pressure, whereas the resting intracellular Ca^2+^ concentration was more consistent in the apical-out organoids. Using this advanced model, we provided evidence about the expression and function of the Ca^2+^ activated Cl^−^ channel Anoctamin 1 (ANO1) and the epithelial Na^+^ channel (ENaC) in the ductal cells. Finally, we demonstrated that the available functional assays, such as forskolin-induced swelling, or intracellular Cl^−^ measurement have improved dynamic range when performed with apical-out organoids. Taken together our data suggest that polarity-switched human pancreatic ductal organoids are suitable models to expand our toolset in basic and translational research.

## Materials and methods

### L-WRN conditioned media

L-WRN cell line was grown in a selection medium containing 10% FBS and 0.5–0.5 mg/ml G418 and Hygromycin B in ATCC-formulated DMEM [23]. The conditioned medium supplemented with 10% FBS and 1–1% kanamycin-sulfate and antibiotic–antimycotic solution has been collected three times from the date of plating every three days and pooled before further applications to minimalize batch-to-batch variation. Materials are listed in Supplementary Table 1.

### Generation of human pancreas organoid cultures and manipulation of cell polarity

Human pancreatic tissue samples were collected from cadaver donors (Ethical approval No.: 37/2017-SZTE). Establishment and maintenance protocols were described previously [23]. Briefly, collected tissue samples were placed in splitting media (Supplementary Table 2) and enzymatic digestion at 37 °C was performed in digestion media (Supplementary Table 3). Efficiency of the tissue digestion was verified by stereomicroscopy every 10 min. The digested cell suspension was centrifuged (200 RCF, 10 min, 4 °C, Rotor radius: 180 mm) and the pellet was washed 3 times in total in wash media (Supplementary Table 4) and resuspended in Matrigel. Next, 10 µL Matrigel was placed in each well of a 24-well cell culture plate and left for 10 min for solidification at 37 °C and 500 µL feeding media was applied in each well (Supplementary Table 5). For culture splitting/subculturing, Matrigel removal and cell separation were performed simultaneously by using a 25 V/V% TrypLE™ Express Enzyme in DPBS at 37 °C for 15–20 min in a vertical shaker followed by two times washing and plating steps as described above. Polarity change of fully grown organoids was induced by extracellular matrix removal with a 7-min-long treatment of previously described digestion media (Supplementary Table 3) at 37 °C followed by two gentle washing steps (parameters of centrifugation: 8 RCF, 10 min, 4 °C, Rotor radius: 180 mm). Organoids with the retained cystic structure were replaced in 6-well plates pre-treated with Anti-Adherence Rinsing Solution and fed with the feeding medium described above. All applied materials are listed in Supplementary Tables 2–6.

### Cryopreservation of primary epithelial cells

For cryopreservation organoids were digested to single-cell suspension by TrypLE™ Express Enzyme as described above. After 3 washing steps (50 RCF, 5 min, 4 °C, Rotor radius: 180 mm) the cell pellet was resuspended in Cryo medium based on feeding media and supplemented with 40% FBS and 5% DMSO. Cells were frozen in a propanol-filled container (Nalgene Mr. Frosty, Sigma, C1562) according to the manufacturer’s protocol and stored in liquid nitrogen long-term. Materials are listed in Supplementary Table 7.

### Gene expression analysis by qRT-PCR

Apical-in and apical-out hPOCs were used for RNA purification by NucleoZOL reagent. RNA concentration and purity of the samples were checked by NanoDrop spectrophotometer. In total, 1 μg purified mRNA was used for cDNA synthesis which was carried out by iScript cDNA Synthesis kit. RT-PCR reactions were performed by SsoAdvanced Universal SYBR Green Supermix and *CFTR* and *ANO1* cDNA-specific primers. Relative gene expression analysis was performed by the ΔΔCq technique. All the applied primers sets and reagents are listed in Supplementary Table 8–9.

### Gene expression analysis of hPOCs by RNA-Seq

RNA was extracted from the collected cell pellet by NucleoSpin RNA Plus kit according to the manufacturer’s protocol (Supplementary Table 10). RNA-sequencing was performed by Illumina NextSeq 500 instrument and data analyses process service were provided by DeltaBio2000 Ltd. Gene expression pattern was determined according to TPM (transcript/million) values (Table 1).

### siRNA transfection

Apical-out hPOCs were transfected with 50 nM siRNA or siGLOGreen transfection indicator and Lipofectamine 2000 in Feeding media (Supplementary Table 5) for 48 h. Pre-designed and validated siRNA sets used for gene-specific knockdown are listed in Supplementary Table 11.

### Immunofluorescent labelling and confocal microscopy

Immunofluorescent labelling on organoid cross sections was performed as previously described [23]. Briefly, organoids were frozen and sectioned in Shandon™ Cryomatrix™ at − 20 °C. Sections (7 μm thick) were placed on microscope slides. Samples were fixed at 4% PFA-PBS for 20 min followed by washing for 3 × 5 min in PBS. Antigen retrieval was performed in Sodium Citrate—Tween20 buffer (0.001 M Sodium Citrate Buffer, pH 6.0 and 0.05% Tween 20) at 94 °C for 30 min followed by a blocking step with 0.1% goat serum and 10% BSA in PBS for 2 h at 37 °C. Primary antibodies were applied overnight at 4 °C while secondary antibodies were incubated for 2 h at room temperature. Nuclear staining and mounting carried out simultaneously by ProLong™ Gold Antifade mounting medium with DAPI. Images were captured by a Zeiss LSM880 confocal microscope using a 40 × oil immersion objective (Zeiss, NA: 1.4). Antibodies and materials applied are listed in Supplementary Table 12.

### Scanning electron microscopy

Organoids were fixed with 2.5% (v/v) glutaraldehyde and 0.05 M cacodylate buffer (pH 7.2) in PBS overnight at 4 °C. 8 μL samples spotted onto a silicon disc coated with 0.01% (w/v) poly-l-lysine. Discs were washed twice with PBS and dehydrated with a graded ethanol series (30%, 50%, 70%, 80%, 100% ethanol (v/v), for 2 h each at 4 °C and 100% EtOH, for overnight). Following dehydration, samples were immersed for 5 min in pure hexamethyldisilazane (HDMS) and air dried. All specimens were mounted on aluminium stubs using double-sided carbon tape and then were sputter coated with 15 nm gold in a sputter coater (Quorum Technologies, Laughton, East Sussex, UK) and observed under a Zeiss Sigma 300 Field-Emission scanning electron microscope (ZEISS). Materials are listed in Supplementary Table 13.

### Fluorescent microscopy and reverse swelling assay

Apical-in and apical-out organoids were attached to poly-l-lysine coated cover glasses and were incubated in HEPES solution for 30 min at 37 °C with 5% CO_2_. BCECF-AM, Fura2-AM (5–5 μmol/L), MQAE (2 μmol/L) and SBFI-AM (10 μmol/L) fluorescent indicators were applied for pH, Ca^2+^, Cl^−^ and Na^+^ measurements, respectively, as previously described [18]. After loading with the fluorescent indicator, the samples were mounted on an Olympus IX73 inverted microscope and were excited with a CoolLED PE-4000 (BCECF-AM) or a CoolLED pE-340fura (Fura2-AM, MQAE and SBFI-AM) illumination system. Filter combinations for each fluorescent dye were described previously [18, 24]. Hamamatsu Orca-Flash 4.0 sCMOS camera and 20X water immersion objective (Olympus; NA: 0.8) with a temporal resolution of 1 s were used for capturing fluorescent signals. Ratiometric image analysis for pH_i_ measurements was performed by Olympus Excellence software. Fluorescence signals of Fura2- and MQAE-loaded samples were normalized and represented as *F*1/*F*0 values. During reverse swelling assay transmission light illumination was applied. Images were taken every minute and were analyzed and quantified by ImageJ software. Reagents and inhibitors applied in experiments are listed in Supplementary Tables 14–15.

### Statistical analysis

Data are expressed as means ± SEM. Significant difference between groups was determined by Mann–Whitney test. *P* < 0.05 was accepted as being significant. All statistical analyses were carried out by GraphPad Prism software (Version 8.3.1).

## Results

### Primary epithelial organoids established from human pancreatic tissue samples retain ductal characteristics and polarity

**Fig. 1:**
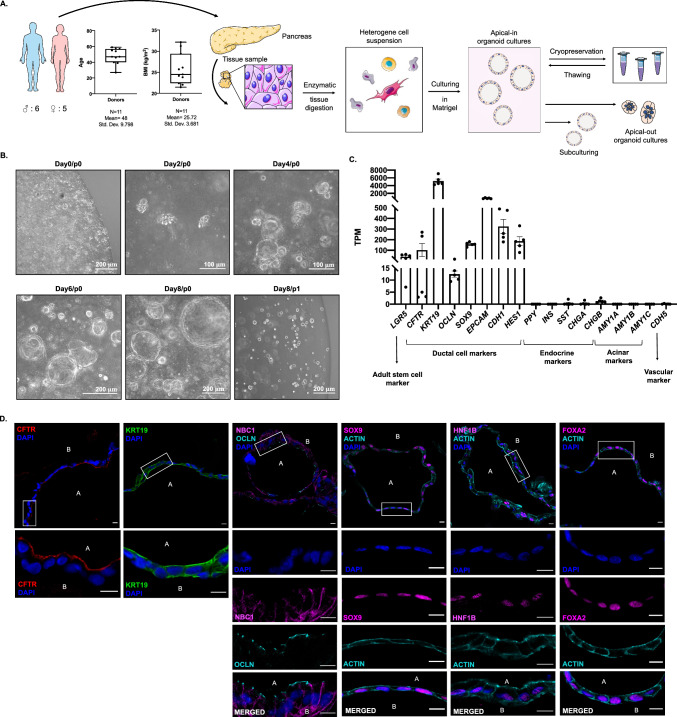
Human pancreatic organoid cultures retain ductal characteristics and apical-to-basal polarity. **a** Schematic overview of sample processing and basic characteristics of the cadaver donors (*N* = 11, mean age: 48 ± 9.798 years and mean BMI: 25.72 ± 3.681 kg/m^2^). **b** Transmitted light images demonstrate growth of human pancreatic organoids from the time of isolation (day 0/passage 0) to first passage (day 8/p1). **c** Transcriptome analysis of human pancreatic organoid cultures (hPOCs) by RNA-sequencing (*N* = 5). Data are indicated in TPM value (transcript/million). Gene abbreviations are in Supplementary Table 16. **d** Confocal images demonstrate the localization CFTR, KRT19, NBC1, OCLN, SOX9, ACTIN, HNF1B and FOXA2 in apical-in human pancreatic organoids (scale bar: 10 μm; A: apical side; B: basolateral side)

Pancreatic tissue samples were collected from 11 cadaver donors with no documented exocrine or endocrine pancreatic disease (6 male and 5 female patients; mean age 48 ± 9.798 years; mean BMI 25.72 kg/m^2^). After mechanical dissociation and enzymatic tissue digestion human pancreatic organoid cultures (hPOCs) were established in ECM (Matrigel) and grown in organoid-feeding media containing Wnt3A/R-spondin/Noggin. Conventional organoids with apical-in polarity were successfully generated from all 11 samples, which were subcultured and cryopreserved in large quantities after the first passaging step for further applications such as creating polarity switched apical-out hPOCs (Fig. 1a). Cystic organoids appeared in the heterogeneous cell suspension on the second day of culture, while erythrocytes, acinar and stromal cells degraded. During passaging the organoids were enzymatically digested into single cells ensuring the clonal growth of the hPOCs (Fig. 1b). Cell fate of the cells was first investigated by RNA-sequencing, where the active expression of the adult stem cell marker *LGR5* (Leucin-rich repeat-containing G-protein coupled receptor 5) and ductal markers such as cystic fibrosis transmembrane conductance regulator (*CFTR)*, cytokeratin 19 (*KRT19)*, occludin (OCLN), SRY-Box transcription factor 9 (*SOX9*), epithelial cell adhesion molecule (*EPCAM*), e-cadherin (*CDH1*), Hes family BHLH Transcription Factor 1 *(HES1*) was observed, while the absence of acinar- (amylase, *AMY1A-C*) endocrine- (Pancreatic polypeptide, *PPY;* Insulin, INS; Chromogranin A-B, (*CHGA-B*) and hematopoietic (vascular endothelial cadherin, C*DH5*) markers confirmed that the human pancreatic organoids containing ductal epithelial cells exclusively (Fig. 1c). Next, we also confirmed the expression and localization pattern of the key ductal markers on the protein level. These ductal markers such as SOX9, HNF1B and FOXA2 showed a typical nuclear localization pattern, whereas KRT19 displayed intracellular reticular expression, which is typical for a cytoskeletal protein. Moreover, the apical-to-basal polarity of the organoids was evidenced by the apical membrane localization of CFTR and OCLN and the basolateral expression of NBCe-1 (Fig. 1d).

### Extracellular matrix removal induces a polarity switch that reduces the epithelial cell tension in pancreatic organoids

**Fig. 2:**
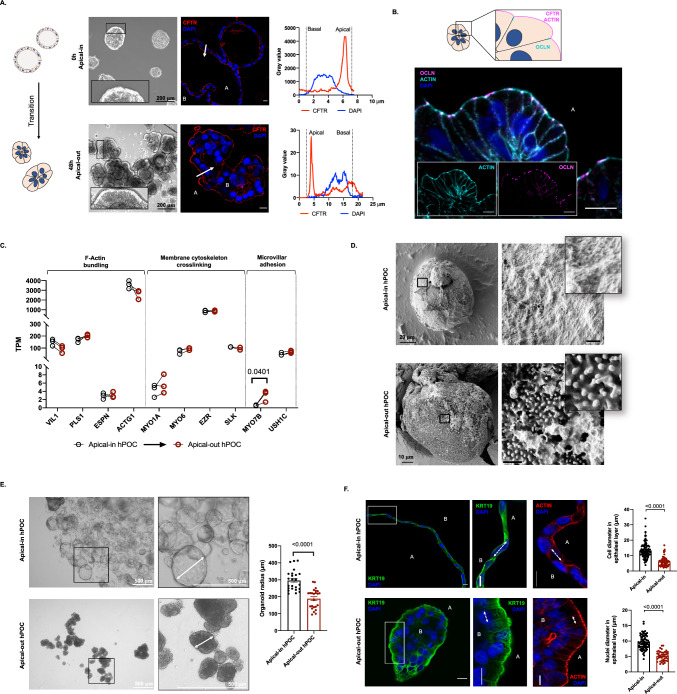
Polarity switching reduces intraluminal tension on the epithelial cell layer of hPOCs.** a** Schematic drawing and transmitted light images demonstrate the 48-h-long transition (polarity switch) in suspension culture. The change of CFTR localization is presented by confocal images and line profile analysis of cross sectioned apical-in and apical-out hPOCs (scale bar: 10 μm; A: apical side; B: basolateral side, white arrow represents the axis of the line profile analysis). **b** The polarity switch was further confirmed by immunofluorescent labelling of Occludin (OCLN) and ACTIN filaments at the apical side (outer side) of the apical-out hPOCs. **c** Transcriptomic comparison of genes responsible for the formation and organization of microvilli in apical-in and polarity switched apical-out hPOCs. Data are indicated in TPM value (Transcript/million). Genes are classified into the following groups based on the function of their protein product: genes responsible for F-actin bundling, membrane cytoskeleton crosslinking and intermicrovillar adhesion. (Gene abbreviations are placed in **Supplementary Table 16**.) **d** Scanning electron microscopy (SEM) pictures of hPOCs revealed brush border and microvilli formation on the outer surface of the apical-out organoids which was not observed on conventional apical-in organoids (scale bars on enlarged images represent 1 μm). **e** Phase contrast images and statistical comparison of the significant diameter difference between apical-in (growing in ECM) and apical-out (suspension culture) hPOCs. **f** Confocal images of crossed sectioned organoids and bar charts demonstrate the significant difference in diameter of cells and nuclei between apical-in or apical-out hPOCs (scale bar: 10 μm; A: apical side; B: basolateral side; white arrows demonstrate the diameter of cells or nuclei)

Previously, Co et al. successfully controlled the polarity of colon organoids by removal of the extracellular matrix scaffold [20, 21], which resulted in a polarity switch of the cells. The apical-out organoids maintained the barrier function allowing the study of host–pathogen interactions. Notably, the epithelial cell functions, such as ion and fluid secretion were not assessed in those manuscripts. To induce a polarity switch in human pancreatic organoids first we generated apical-in organoids in ECM, which then were placed into a suspension culture. We observed that after 48 h the morphology of the organoids was changed and the typical cystic form was replaced by a denser structure formed by a columnar cell layer (Fig. 2a). Immunofluorescence staining of the apical CFTR, ACTIN and OCLN revealed that the morphological changes were also followed by the redistribution of intracellular proteins (Fig. 2a, b). Previously we demonstrated the presence of a brush border on the luminal membrane of mouse pancreatic organoids, which can promote epithelial secretion by increasing the membrane surface [18]. To further decipher the effect of ECM removal on the organoids, we determined the expression of genes participating in microvillus formation and maintenance [25, 26]. Our results showed that there were no significant differences in the expression of genes involved in F-actin bundling, membrane cytoskeleton crosslinking or intermicrovillar adhesion upon polarity switching (Fig. 2c). However, the expression of myosin 7B was significantly higher in apical-out organoids. These transcriptomic data suggest that the polarity change may increase the formation of microvilli and the brush border. In addition, scanning electron microscopy revealed the formation of a dense brush border on the outer surface of the polarity-switched human pancreatic organoids (Fig. 2d). The apical-in organoids display vectorial ion and fluid secretion into the lumen [18, 27] resulting in an elevated intraluminal pressure and cyst formation, which may also affect the cell shape. Therefore, we examined the size of the organoids before (in ECM) and after (in suspension) the polarity change. We found that the diameter of the organoids significantly decreased, whereas the cell density increased after the polarity switch (Fig. 2e). Next, we compared the diameter of cells and nuclei in the apical-in and apical-out hPOCs, which revealed that the longitudinal diameter of the cells and nuclei significantly reduced in apical-out organoids leading to the formation of a columnal epithelial cell layer (Fig. 2f). In epithelial cells, PIEZO1 function as a mechanosensor mediating extracellular Ca^2+^ influx upon increased intraluminal tension [19]. Our results also showed that hPOCs express *PIEZO1* (Supplementary Fig. 1A-B.) that can participate in the regulation of epithelial ion and fluid secretion. Finally, we showed that the polarity switching decreases the gene expression of cyclins, which are major elements of cell division, suggesting a decrease in cell proliferation. Our results highlight that the removal of the ECM can induce a polarity switch in the hPOCs that eliminates the intraluminal pressure leading to a columnar cell morphology, which can be observed in primary ductal cells in their physiological environment [18].

### The resting intracellular Ca^2+^ concentration is more consistent in apical-out hPOCs

**Fig. 3:**
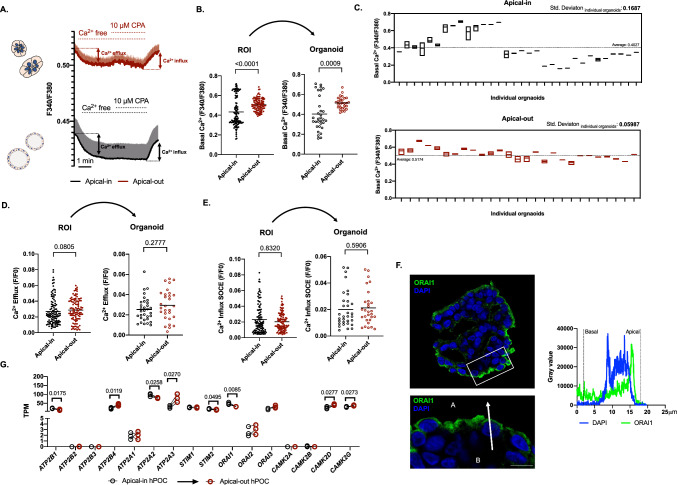
The resting intracellular Ca^2+^ concentration is more consistent in apical-out hPOCs. **a** Average traces demonstrate the resting Fura2-AM ratio. Removal of the extracellular Ca^2+^ triggered a drop of intracellular Ca^2+^ presumably due to cytosolic Ca^2+^ efflux. 10 μM CPA (cyclopiazonic acid) was administrated in Ca^2+^ free HEPES for ER Ca^2+^ depletion, whereas re-addition of the extracellular Ca^2+^ initiated store-operated Ca^2+^ entry (SOCE). **b** Bar charts show the significant difference in basal Ca^2+^ levels between apical-in and apical-out organoids based on the investigated ROIs which was also converted into individual organoids. **c**Graphs demonstrate the standard deviation of basal Ca^2+^ levels in individual organoids from the mean value. **d**, **e** Bar charts show a maximum change in Ca^2+^ levels during Ca^2+^ efflux and CRAC channel activity (Ca^2+^ influx) with no significant differences in apical-in and apical-out hPOCs. **f** Confocal images and line profile analysis demonstrate the apical localization of ORAI1 on cross-sectioned apical-out hPOCs (scale bar: 10 μm; A: apical side; B: basolateral side, the white arrow represents the axis of the line profile analysis). **g** Comparative (apical-in/apical-out) transcriptomic analysis of major genes responsible for Ca^2+^ handling. Data are indicated in TPM value (transcript/million). Gene abbreviations are placed in Supplementary Table 16

Ca^2+^ signaling is one of the major signal transduction pathways regulating secretory processes in the exocrine pancreas whereas in pathophysiological conditions, inflammatory processes are always characterized by disturbed Ca^2+^ homeostasis [6, 28]. Since the above-described intraluminal tension in the apical-in culture may influence the epithelial Ca^2+^ homeostasis via PIEZO1 activation, we compared the Ca^2+^ signaling in apical-in and apical-out hPOCs. Evaluation of the resting intracellular Ca^2+^ levels revealed a significantly elevated basal Ca^2+^ level in apical-out hPOCs compared to apical-in organoids (Fig. 3a, b). On one organoid multiple ROIs were selected during the measurement. To translate the signals detected in ROIs on organoid level, all ROIs selected on an individual organoid were pooled and plotted. In both cases, the basal intracellular Ca^2+^ levels were significantly higher in the apical-out organoids (Fig. 3b). Interestingly, if each organoid was plotted separately with the mean value of all ROIs, it is clearly shown that apical-in organoids have 2.8 times higher basal Ca^2+^ level deviation than their apical-in counterparts (Fig. 3c). These observations suggest that the resting intracellular Ca^2+^ is more constant in the apical-out hPOCs. Notably, no significant difference was found in either the Ca^2+^ efflux or Ca^2+^ influx rates in a similar evaluation process (Fig. 3d, e), suggesting that the observed deviation in the basal intracellular Ca^2+^ in the apical-in organoids may be a result of a pre-stimulated state (such as the activation of PIEZO1 by mechanical stress), which varies among different organoids. Since it was previously shown that ORAI1 CRAC channel is a promising therapeutic target to prevent epithelial cell Ca^2+^ overload we also investigated the channel localization in apical-out hPOCs which retained its apical membrane expression (Fig. 3f) [24]. To rule out that the changes observed in the intracellular Ca^2+^ homeostasis were caused by the altered expression of proteins involved in the Ca^2+^ signaling, we analyzed and compared the expression of several genes (Fig. 3g.). However, no biologically meaningful changes were observed between apical-in and apical-out organoids. Taken together, our data suggest that resting intracellular Ca^2+^ level is more consistent in the apical-out hPOCs, which may be explained by the elimination of the intraluminal pressure and no pre-stimulation in this culture.

### Functionally active Anoctamin 1 and ENaC are expressed in human pancreatic ductal cells

**Fig. 4:**
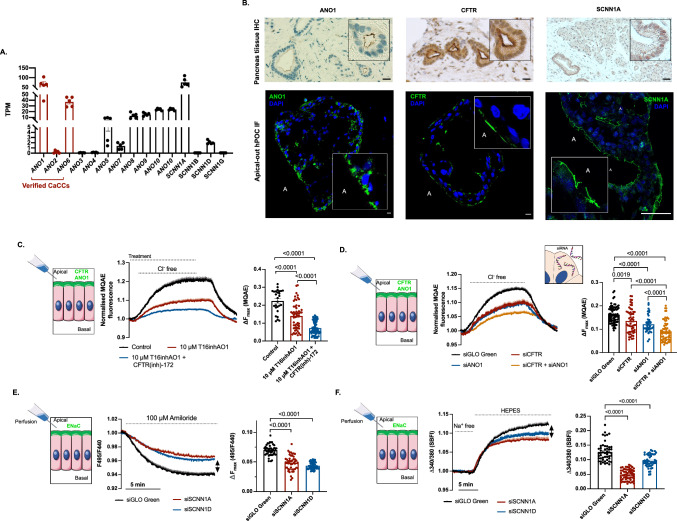
Polarity switching revealed the role ANO1 and ENaC in the apical ion secretion in human pancreatic ductal cells. **a** Gene expression profile of *ANO* family members and *SCNN1A/B/D/G* (Gene abbreviations are placed in Supplementary Table 16.) in apical-in hPOCs. Data are indicated in TPM value (Transcript/million). **b** Comparison of IHC performed on sectioned pancreatic tissue and IF-labeled apical-out hPOC to demonstrate retained apical localization of ANO1, CFTR and SCNN1A (scale bar: 10 μm; A: apical side). **c** Average traces and bar charts demonstrate the intracellular Cl^−^ level measurements performed by MQAE in apical-out organoids. The intracellular [Cl^−^]_i_ and the intensity of fluorescence are inversely proportional. Challenging apical-out organoids with Cl^−^ free extracellular solution resulted in a decrease in [Cl^−^]_i_ which was significantly reduced by 10 μM of ANO1 inhibitor (T16inhAO1) while in combination with 10 μM CFTR(inh)-172 the mean maximum response was almost completely abolished. **d** Average traces and bar charts demonstrate the result of siRNA transfection by 50 nM siGLO Green (control), si*CFTR*, or si*ANO1* in the same experimental setup. Both siRNAs, alone and in combination significantly reduced Cl^−^ efflux compared to the control. **e** Average traces and bar charts demonstrate a significant difference in (BCECF-AM) fluorescent ratio between siGLO Green (control) and si*SCNN1A* or si*SCNN1D* treated apical-out hPOs in response to 100 μM amiloride. Average traces of 3–4 experiments are demonstrated in each measurement. **f** Average traces and bar charts demonstrate a significant difference in (SBFI) fluorescent ratio between the control and siRNA treated apical-out hPOs in response to Na^+^ free solution Average traces of 3–4 experiments are demonstrated in each measurement

Pancreatic organoids enabled the direct study of human pancreatic secretory functions. According to the currently accepted model CFTR plays a central role in the human pancreatic ductal Cl^−^/HCO_3_^−^ secretion, whereas other Cl^−^ channels, such as Ca^2+^ activated Cl^−^ channels (CaCC) are not expressed in the ductal cells, or have marginal role [29–32]. In contrast in other species, such as rodents, CaCC are important in pancreatic ion secretion [33]. These early pharmacological studies were also confirmed by RNA-sequencing, which revealed that *Ano1* is the dominantly expressed CaCC channel in apical-in mouse pancreatic organoids (Supplementary Fig. 2A), which is 45.73 times higher than *Cftr* (Supplementary Fig. 2B). To gain human-relevant information, we also analyzed the expression of the anoctamins (ANO) and the epithelial sodium channel (ENaC) subunits, which was not found previously in the pancreatic ductal cells [36], in human pancreatic organoids. Interestingly, transcriptome analyses revealed a relatively high expression of *ANO1* and *SCNN1A* in the apical-in human pancreatic organoids (Fig. 4a). The average expression of *CFTR* and *ANO1* in the human pancreatic organoids is nearly identical suggesting that ANO1 as the dominantly expressed ANO family member may contribute to the ion secretion in human ductal cells (Supplementary Fig. 2C). To confirm these findings in human pancreatic tissue, we next demonstrated the expression of ANO1 and ENaC by immunohistochemistry carried out on the human pancreas tissue section and by immunofluorescent labelling performed on apical-out hPOCs (Fig. 4b). The observed pattern suggests that also ANO1 and ENaC are dominantly present in the apical membrane of the ductal epithelial cells. When we assessed the effect of the polarity switch on the gene expressions, we observed that *CFTR* is not affected by this process, while the expression of *ANO1* was significantly decreased suggesting its stronger dependence on intraluminal pressure (Supplementary Fig. 2D). Next, we used an intracellular Cl^−^ concentration ([Cl^−^]_i_) sensitive fluorescent indicator, MQAE to follow ANO1 driven Cl^−^ extrusion. Due to the chemical characteristics of MQAE, the emitted fluorescent signal inversely correlates with [Cl^−^]_i_. Apical-out hPOCs were challenged with 10 μM T16inhAO1, a pharmacological inhibitor of ANO1, for 5 min before the extracellular Cl^−^ removal from the HCO_3_^−^/CO_2_^−^buffered solution. These experiments revealed that 10 μM T16inhAO1 significantly reduced the intracellular Cl^−^ extrusion. Moreover in combination with 10 μM CFTR(inh)-172 the maximum response was further reduced (Fig. 4c). Direct application of siRNA to knock down gene expressions in organoids is hindered by the presence of Matrigel, which limits the cell permeation of siRNAs (Supplementary Fig. 3.). However, this bottleneck was eliminated by culturing the organoids in suspension, which allowed the knock down of CFTR and ANO1 expression in apical-out organoids as verified by qRT-PCR, immunofluorescence staining and intensity profile analysis of the target proteins (Supplementary Fig. 4A-B). These experiments demonstrated that both siCFTR and siANO1 significantly decreased the apical Cl^−^ extrusion, which was further impaired by the combination of the two siRNAs (Fig. 4.D.). These results provide evidence of the significant contribution of ANO1 to the Cl^−^ secretion of human pancreatic ductal cells, which may have a major and yet unrevealed physiological relevance.

ENaC is usually expressed on the apical plasma membrane of the epithelial cells and participates in Na^+^ reabsorption [34, 35]. ENaCs are heterotimers that are formed by α, β, and γ subunits (with a 1:1:1 stoichiometry) encoded by different genes (*SCNN1A, SCNN1B, SCNN1G*) [37, 38]. Another subunit known as δ (*SCNN1D)* has been identified in humans [39]. It was previously shown that functional ENaC formed by single or only two subunits, exists in certain tissues but the exact structure and capacity of the channel in pancreatic ductal cells remained unexplored [40]. Interestingly, among 4 subunits, remarkable expression of *SCNN1A* and moderate expression of *SCNN1D* were detected in hPOCs (Fig. 4a). Moreover, the subunit α (SCNN1A) was detected in the apical membrane of polarity-shifted hPOCs by immunofluorescent labelling (Fig. 4b). Next, siRNA interference was applied to assess the functional activity of the channel. After 48 h incubation with siSCNN1A and siSCNN1D, apical-out hPOCs were challenged with 100 μM amiloride from the apical membrane, which resulted in a rapid decrease of the fluorescent ratio suggesting an indirect influence of the ENaC activity on the apical Cl^−^/HCO_3_^−^ exchange (Fig. 4e). Notably, to achieve the significant decrease in the maximal response, the knockdown of each subunit was sufficient suggesting that both isoforms are needed for the formation of the active channel. Furthermore, the intracellular Na^+^ indicator SBFI was used to directly measure the contribution of SCNN1A and SCNN1D to the channel activity, which further confirmed that both subunits are mandatory for the function of ENaC as both siRNA treatment resulted in significantly reduced Na^+^ influx (Fig. 4f) These results suggest that SCNN1A and SCNN1D subunits are necessary for the constitution of a functional ENaC proteins in human pancreatic ductal cells.

### Apical-to-basal polarity switch improve the performance of organoids in available functional assays

**Fig. 5:**
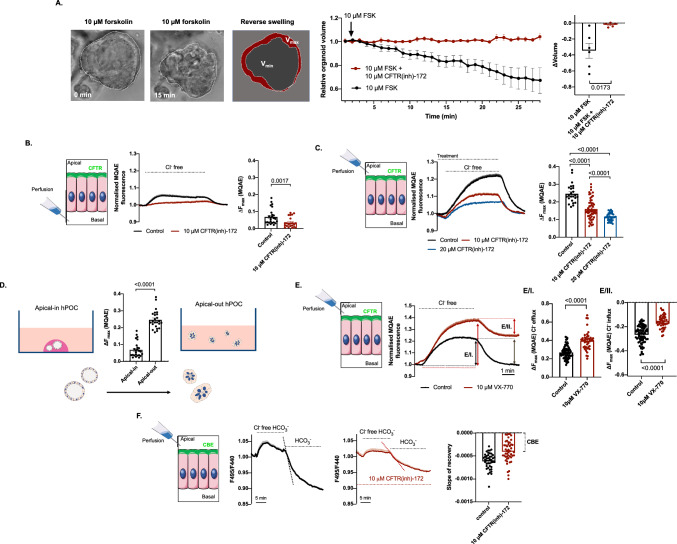
Apical Cl^−^ efflux is enhanced in apical out hPOCs. **a** Transmitted light images and the relative organoid volume demonstrate the change of organoids during inverse swelling assay induced by 10 μM Forskolin (FSK). Relative change of organoid volume was normalized to the baseline state. 10 μM CFTR(inh)-172 completely abolished the volume change. **b** Intracellular Cl^−^ level was measured by MQAE in apical-in organoids. Challenging apical-in organoids with Cl^−^ free extracellular solution resulted in a decrease in [Cl^−^]_i_ which was abolished by 10 μM CFTR(inh)-172. Average traces and bar charts demonstrate the significant difference between the investigated groups (control vs 10 μM CFTR(inh)-172). **c** Elevated Cl^−^ extrusion observed in apical-out hPOs could not be completely abolished by 10 or 20 μM CFTR(inh)-172. **d** The bar chart demonstrates the comparison of detected maximum Cl^−^ extrusion of apical-in vs apical-out hPOCs. **e** Average traces and bar charts show the effect of the CFTR-potentiator VX-770 which significantly enhanced Cl^−^ efflux (**e/I**) and which also established an elevated equilibrium in [Cl^−^]_i_ level upon reperfusion of the Cl^−^ containing solution (**e/II) f** Intracellular pH was used for the detection of Cl^−^/HCO_3_^−^ exchanger (CBE) activity. Average traces and bar charts demonstrate the significant difference between slopes in control vs 10 μM CFTR(inh)-172 treated groups at post-withdrawal phase of the extracellular Cl^−^. The difference between the mean slope values indicates CBE activity. Average traces of 3–4 experiments are demonstrated in each measurement

To use of ex vivo models with translational relevance and improved throughput is currently an unmet need in pancreatic research. As an example, current drugs that are accepted for the treatment of cystic fibrosis have been designed to rescue the lung phenotype of CF, however, the pancreatic phenotype have not been addressed yet. This is most likely due to the lack of access to functional human pancreatic cells. To assess CFTR activity in epithelial cells a widely used indirect functional assay is the forskolin-induced swelling (FIS) [41]. However, the dynamic range of this technique is limited by the elevation of the intraluminal pressure within the organoids. By capitalizing the switched polarity of apical-out organoids, we performed a reverse FIS assay to demonstrate the activity of the wild-type CFTR. Under these experimental conditions 10 µM forskolin (FSK) decreased the relative volume of the apical-out organoids, which was abolished by the administration of 10 µM CFTR(inh)-172 (Fig. 5a) suggesting that this technique is suitable to measure the activity of the wild type CFTR as well. Next, we wanted to compare further the performance of the apical-in and apical-out organoids in functional assays, therefore, we assessed CFTR-mediated Cl^−^ secretion by MQAE. The Cl^−^ extrusion was significantly decreased by 10 µM CFTR(inh)-172 suggesting that the detected change is largely CFTR dependent in apical-in organoids (Fig. 5b, c). However, when the same protocol was applied to apical-out organoids, the response to Cl^−^ removal was higher than in conventional organoids presumably caused by the direct apical perfusion and lack of intraluminal pressure (Fig. 5c, d). Moreover, due to the enhanced resolution of the apical-out model, even the administration of a 20 µM CFTR(inh)-172 inhibitor could not completely abolish the Cl^−^ efflux process, confirming the involvement of additional channels, such as ANO1 (Fig. 5c). The direct access of the apical membrane also allowed us to test the effect of a known CFTR potentiator, VX-770 on the CFTR activity on the pancreatic ductal cells. The administration of 10 μM VX-770 significantly enhanced Cl^−^ efflux in apical-out organoids and kept the steady-state at an elevated [Cl^−^]_i_ proving proof-of-concept evidence for the applicability of apical-out hPOCs in in vitro drug testing assays (Fig. 5e/eI–II.). Finally, Cl^−^ and HCO_3_^−^ exchanger (CBE) CBE activity was measured by using BCECF pH-sensitive dye with CFTR inhibition in HCO_3_^−^/CO_2_ buffered solution. Cl^−^ withdrawal followed by direct apical perfusion of Cl^−^ containing HCO_3_^−^ solution with simultaneous administration of 10 μM CFTR(inh)-172 decreased the slope of BCECF ratio (F495/F440) compared to the control group, which difference suggests indirect detection of CBE activity in hPOCs (Fig. 5f). These data suggest that apical-out hPOCs could provide a significantly improved platform for ex vivo functional assays and pharmacological tests with increased throughput.

## Discussion

In this manuscript, we generated human pancreatic organoids and advanced the culture technique further by manipulating the polarity of the epithelial cells. By switching apical-to-basal polarity the elongated cell morphology observed in the apical-in organoids changed to a cuboidal shape in the apical-out cultures, which was accompanied by a more consistent resting intracellular Ca^2+^ level. Capitalizing on the improved accessibility of the apical plasma membrane, we identified the expression and function of ANO1 and ENaC in the human ductal epithelial cells. Finally, we demonstrated that functional assays (such as FIS, or CFTR activity measurements) display an improved dynamic range when performed using apical-out organoids.

In recent years, organoid cultures derived from tissue-specific Lgr5+ adult stem cells emerged as novel models of organ development and disease [10, 11]. By maintaining the activity of Wnt/β-Catenin signal transduction cascade—a key driver of most types of adult stem cells [12]—organoid cultures (OCs) can be grown in vitro for long-term in 3D extracellular matrix-based hydrogels; whereas, epithelial cells in the culture maintain the original cellular diversity and organization of the organ of origin [13]. Originally small intestinal adult stem cells were used to generate crypt–villus like structures in vitro [14]. Since then OCs have been established from a wide range of organs in the gastrointestinal tract, including colon, esophagus [10], stomach [15], liver and pancreas [16]. Our group previously provided morphological and functional comparison of primary epithelial cells in mouse-isolated ductal fragments and pancreatic organoid cultures. We demonstrated that the apical-to-basal polarity of the epithelial cells, gene and protein expressions and ion transport activities in the mouse pancreatic organoids remarkably overlap with those observed in freshly isolated primary ductal fragments [18]. These results highlighted that organoids are suitable to study epithelial cell functions as well. The previously published pancreatic organoid establishment protocol was based on the manual picking of pancreatic ductal fragments after digesting the pancreatic tissue [42]. This step eventually requires manual work of pancreatic ductal fragment isolation and experience; which limits the throughput and hinder the automatization of the workflow. One significant innovation of the current study is the replacement of the manual picking of individual ductal fragments with an improved enzymatic digestion protocol of the whole pancreatic tissue, which improved the yield and success rate of organoid generation. The generated pancreatic organoids consisted solely of polarized epithelial cells and the success rate was 100%. The epithelial cells in the OCs expressed well-known ductal markers like CFTR, KRT19, SOX9, HNF1B or FOXA2, whereas non-ductal markers like amylase or insulin were completely absent. This suggests that our advanced technique is suitable to generate pure pancreatic ductal cell cultures with high efficiency without the presence of other cell types. Another potential model to study pancreatic diseases is the human-induced pluripotent stem cells (hiPSCs) based organoids provide a unique platform for developmental studies and regenerative medicine [43]. The generation of pancreatic progenitor cells from PSCs is based on a sequential induction of definitive endoderm, foregut endoderm and pancreatic endoderm [44]. Although the differentiation of PSCs towards endocrine pancreatic progeny has been published [45], the generation of ductal and exocrine-like cells has not yet been adequately achieved. In a recent study, Hohwieler et al. developed a novel differentiation protocol and successfully generated human pancreatic acinar/ductal organoids from controls and cystic fibrosis patients that also recapitulated the defective CFTR function [46]. The cells in this culture also expressed acinar cell markers, such as amylase suggesting that it was a mixture of acinar and ductal cells. Recently the differentiation protocol was further developed, which improved the presence of pancreatic duct-like organoids [27]. The advantage of the currently published protocol is that it can provide pure ductal organoid cultures within a relatively short timeframe. Moreover, the elimination of the manual steps from the protocol may allow the automatization of the workflow for large-scale manufacturing.

Another well-known limitation of the use of organoids is the ECMs such as Matrigel, which is essential for the growth of organoids, however, significantly reduces the applicable molecular biology toolset. ECM can limit the diffusion of the test compounds and pharmacons due to the molecular size and charge specificity of the individual molecules, whereas it hinders the application of siRNAs and plasmids. In addition, in the conventional organoid cultures the apical membrane of the epithelial cells is not directly accessible for drugs. Moreover, due to the vectorial transport of ions and fluid [9] the intraluminal pressure of the apical-in organoids is elevated without any pre-stimulation, which may affect the epithelial secretory processes [47]. To overcome these limitations, we induced the switch of the apical-to-basal polarity of the organoids by replacing the established organoids with an ECM-free suspension culture. Recently, Co et al. developed a technique to reverse enteroid polarity to study host–pathogen interactions [20]. They showed that apical-out enteroids maintain apical-to-basal polarity and barrier function in ECM-free media, differentiate into the major intestinal epithelial cell types, and exhibit polarized absorption of nutrients, whereas bacterial entry mimics the in vivo entry process. The authors also demonstrated that the regulation of cell polarity by the ECM highly depends on the interaction of the ECM with β1 integrin, which acts as a receptor for ECM proteins. When the enteroids were treated with β1 integrin function-blocking antibody the polarity of BME-embedded enteroids reversed to an apical-out orientation. Notably, we cannot rule out other ECM-β1 integrin-independent factors in the case of the pancreatic organoids, such as increased intraluminal pressure. In our study immunostaining of CFTR, actin and occluding and visualization of the brush border on the outer surface of the apical-out organoids by scanning electron microscopy revealed a complete switch of the polarity after 48 h. This switch also led to a change of the epithelial cell morphology from an elongated to a cuboidal shape, suggesting that the elimination of the intraluminal pressure also affects cell homeostasis. As an example, we demonstrated that the resting intracellular Ca^2+^ levels in unstimulated apical-out organoids were more consistent, compared to the apical-out organoids, whereas the intracellular Ca^2+^ release or extrusion was not changed. This finding may have a significant impact on the application of pancreatic organoids in different investigations, as the intracellular Ca^2+^ signaling determines the physiological secretion and pathological functions of the ductal cells [48]. It is important to emphasize that sustained elevation of the intracellular Ca^2+^ concentration leads to mitochondrial damage and impaired secretion of the ductal cells [49]. Notably, this property of the apical-out organoids may eliminate the interference with the detectable effect of pharmacons acting on the intracellular Ca^2+^ signaling making this culture a preferential choice for such experiments. We also demonstrated that ductal epithelial cells express the mechanosensitive receptor Piezo1, which is a Ca^2+^ channel and senses the intraluminal pressure and stretch. PIEZO1 is also responsible for the induction of rapid epithelial cell division when it senses mechanical stretching thus regulating epithelial turnover, such as that caused by intraluminal pressure observed in apical-in organoids, which appears to be attenuated by polarity switching [50]. This was also confirmed by the decreased expression of the genes related to the cell cycle in the apical-out organoids. Moreover, Piezo1 also plays a critical role in transducing mechanical signals into an intracellular inflammatory cascade leading to tissue inflammation [51]. The activation of Piezo1 can regulate epithelial barrier functions negatively through Claudin-1 and induce epithelial dysfunction [19]. Notably, the polarity switch induced an increase in the expression of *MYO7*. Since *MYO7* is essentially responsible for the interaction between microvilli, its increased expression is presumably related to a decrease in luminal space and pressure, which could also account for the previously observed sparser microvilli density and size in mouse organoids compared to the patterns seen in the pressure draining tube-like primary isolated ducts [18]. Thus, the presence of intraluminal pressure in cystic organoids may therefore be a factor to be eliminated when comparing physiological and inflammatory conditions.

**Fig. 6:**
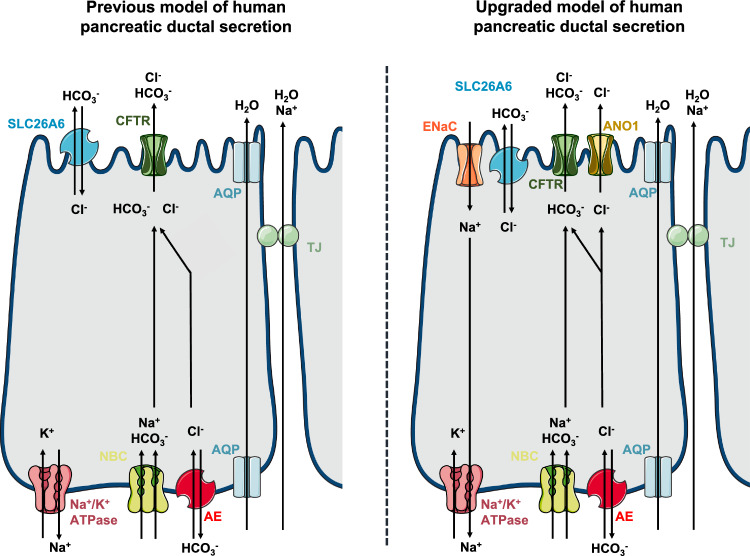
Comparison of the previous and our upgraded model of human pancreatic ductal cell secretion supplemented by novel findings reported in the current paper. Anion exchanger (AE), aquaporin (AQP), tight junction (TJ)

Next, we utilized the developed culture technique and the accessibility of the apical membrane to investigate the ion transport of the polarized human pancreatic ductal cells. The current model of pancreatic ductal HCO_3_^−^ secretion is reviewed in detail elsewhere [1, 52]. Briefly, the secretion of a large amount of HCO_3_^−^ depends on the interaction of the SLC26A6 Cl^−^/HCO_3_^−^ exchanger and the CFTR Cl^−^ channel. SLC26A6 works with a 2HCO_3_^−^:1Cl^−^ stoichiometry, moreover, the STAS domain of the SLC26A6 interacts with the R domain of CFTR leading to increased activation [10, 53, 54]. In addition, stimulation of the cells with cAMP agonists leads to an increased HCO_3_^−^ permeability of CFTR and a marked drop of the intracellular Cl^−^ concentration [55]. This activates the WNK1-OSR1/SPAK pathway, that ultimately led to the phosphorylation of CFTR and a marked increase in the relative permeability of CFTR to HCO_3_^−^ [56]. In contrast to these, using our novel tool, we identified two ion channels that are expressed on the plasma membrane of the ductal cells and were never considered in pancreatic ion secretion. Our results revealed that ductal cells express functionally active ANO1, which is a Ca^2+^ activated Cl^−^ channel. ANO1 expression was only reported on the apical membrane of pancreatic acinar cells, in which HCO_3_^−^ secretion via ANO1 attenuated the pH shift during acute pancreatitis [57]. In addition, we also demonstrated the expression and functional activity of ENaC on the apical membrane of the ductal cells. This was rather surprising, as researchers in the field agree, that ENaC does not participate in pancreatic ductal functions, whereas it is widely expressed in many types of epithelial cells [58, 59], mainly contributing to the reabsorption of luminal Na^+^ and regulation of the volume and composition of the luminal fluid. Previous patch-clamp studies on isolated rat ductal fragments showed no measurable effect of amiloride on ductal cells [36], which was not revised later in other species. Notably, our observations have potential limitations, that need to be considered when we interpret to the physiological role of ENaC in the pancreas. First, pancreatic ductal cells located at the different parts of the ductal tree may exhibit different secretory activities. In the currently used model, we have no exact information on which part of the ductal tree is represented in the organoid culture. In addition, the participation of these channels in the physiological secretory processes needs further evaluation. On the other hand, the introduction of two novel ion channels will require a detailed revision of the secretory process, that will remarkably improve the current model and should lead to a better understanding of the human exocrine pancreatic ion secretion (Fig. 6). As both ENaC [59] and ANO1 [60] are considered potential therapeutic targets in CF and there is a significant interest to develop drugs that modify the channel function, these channels could also be capitalized in the treatment of exocrine pancreatic diseases, such as CF-related exocrine pancreatic insufficiency, or chronic pancreatitis [61, 62].

Finally, we provided examples, how this novel apical-out culture system could be used to improve the currently available functional assays. We demonstrated that the elimination of the intraluminal pressure remarkably improves the dynamic range of the FIS assay. FIS assay on human rectal organoids is used to predict the drug response of CF patients [63]. In these screening assays researchers use rectal biopsy samples obtained from CF patients to generate organoid cultures to study the effect of CFTR correctors and predict the drug response of the patients [64], whereas it may not be suitable in patients with remaining CFTR function [65]. The advantage of our approach is twofold, first, the elimination of the ECM could make the assay suitable for automatization and large-scale screening, second the improved dynamic range may lead to a better resolution of the results and higher precision of the clinical predictions. In addition, we also demonstrated that intracellular Cl^−^ measurements on apical-out organoids provide a better dynamic range and an improved dose–response of the ductal epithelial cells.

Taken together the results presented here go far beyond our current conclusions. By eliminating the intraluminal pressure in conventional organoids, we have not only gained information about the real capabilities of apical channels but also opened the door to multi-field translational research. Polarity-switched human pancreatic organoids offer new options for regenerative therapies for diabetes, acute or chronic pancreatitis or for cystic fibrosis of the pancreas.

## Supplementary Information

Below is the link to the electronic supplementary material.

Supplementary file1 (DOCX 6505 KB)

Supplementary file2 (XLSX 21 KB)

## Data Availability

The datasets generated during and/or analyzed during the current study are fully available upon contact with the corresponding author.
