# Supplementary material for: Human pancreatic ductal organoids with controlled polarity provide a novel ex vivo tool to study epithelial cell physiology
Source: Cell Mol Life Sci. 2023 Jun 28;80(7):192. doi: 10.1007/s00018-023-04836-2 (PMC10307727; doi:10.1007/s00018-023-04836-2)
Supplement: Supplementary file 1 — Supplementary file1 (DOCX 6505 KB) [file 18_2023_4836_MOESM1_ESM.docx]

**Supplementary Table 1.**

| L-WRN conditioned media | | |
| --- | --- | --- |
| Component | Manufacturer | Cat.No. |
| L-WRN cell line | ATCC | ATCC-CRL-3276 |
| FBS | Gibco | 10500064 |
| G-418 | Gibco | 11811031 |
| Hygromycin B | Invitrogen | 10687010 |
| ATCC-formulated DMEM | ATCC | ATCC-30-2002 |
| Kanamycin Sulfate | Gibco | 15160054 |
| Antibiotic-Antimycotic (100X) | Gibco | 15240062 |

**Supplementary Table 2.**

| Generation of human pancreas organoid cultures and manipulation of cell polarity | | |
| --- | --- | --- |
| Spitting media | | |
| Component | Manufacturer/Cat.No. | Final cc/volume |
| Advanced DMEM/F-12 | Gibco, Catalog No.: 12634-010 | 500 ml |
| 1 M HEPES | Gibco, Catalog No.: 15630080 | 5 ml  (10 mM) |
| GlutaMax Supplement(100X) | Gibco, Catalog No.: 35050061 | 5ml  (1X) |
| Primocin (400X) | Invivogen, Catalog No.: ant-pm-2 | 1,25 ml  (1X) |

**Supplementary Table 3.**

| Generation of human pancreas organoid cultures and manipulation of cell polarity | | |
| --- | --- | --- |
| Digestion Media | | |
| Component | Manufacturer/Cat.No. | Final cc/volume |
| Splitting media | - | 19,0984 ml |
| Collagenase IV. | Worthington, Catalog No.: LS004188 | 1250 U/ml |
| Dispase | Sigma-Aldrich, Catalog No: D4693-1G | 0,5 U/ml |
| FBS | Gibco, Catalog No: 10500064 | 0,5 ml  2,5% |
| Soybean Trypsin Inhibitor | Sigma-Aldrich, Catalog No: T9128-1G | 1mg/ml |
| Kanamycin Sulfate | Gibco, Catalog No: 15160054 | 200 µl  1X |
| Antibiotic-Antimycotic Solution | Gibco, Catalog No: 15240062 | 200 µl  1X |
| Voriconazole (25mg/ml) | Tocris, Catalog No: 3760/10 | 1,6 µl  (cc:2 µg/ml) |

**Supplementary Table 4.**

| Generation of human pancreas organoid cultures and manipulation of cell polarity | | |
| --- | --- | --- |
| Wash Medium | | |
| Component | Manufacturer/Cat.No. | Final cc/volume |
| Splitting media | - | 47,7484 ml |
| FBS | Gibco, Catalog No: 10500064 | 1,25 ml (2,5%) |
| Kanamycin Sulfate | Gibco, Catalog No: 15160054 | 500 µl (1X) |
| Antibiotic-Antimycotic Solution | Gibco, Catalog No: 15240062 | 500 µl (1X) |
| Voriconazole (25mg/ml) | Tocris, Catalog No: 3760/10 | 4 µl  (cc:2 µg/ml) |

**Supplementary Table 5.**

| Generation of human pancreas organoid cultures and manipulation of cell polarity | | |
| --- | --- | --- |
| Feeding Medium | | |
| Component | Manufacturer/Cat.No. | Final cc/volume |
| Splitting media | - | 22,808 ml |
| A-83 | TOCRIS, Catalog No: 2939 | 500 nM  (50 µl) |
| EGF | Gibco, Catalog No: PMG8041 | 50 ng/ml  (5 µl) |
| FGF | Gibco, Catalog No: PHG0360 | 100 ng/ml  (5 µl) |
| Gastrin I | TOCRIS, Catalog No: 3006 | 0.01 µM  (5 µl) |
| N-acetylcysteine | Sigma-Aldrich, Catalog No: A9165-56 | 1.25 mM  (125 µl) |
| Nicotinamide | Sigma-Aldrich, Catalog No: NO636-100G | 10 mM  (500 µl) |
| B-27 Supplement  (50X) | Gibco, Catalog No: 17504001 | 1X  (1ml) |
| L-WRN conditioned media | - | 25 ml |
| Y-27632 Rho-Kinase Inhibitor | TOCRIS, Catalog No: 1254 | 10.5 µM  (50 µl) |
| Prostaglandin E2 (PGE2) | TOCRIS, Catalog No: 2296 | 1 µM  (50 µl) |
| Kanamycin Sulfate | Gibco, Catalog No: 15160054 | 1X  (500 µl) |
| Antibiotic-Antimycotic Solution | Gibco, Catalog No: 15240062 | 1X  (500 µl) |
| Voriconazole (25mg/ml) | Tocris, Catalog No: 3760/10 | 4 µl  (cc:2 µg/ml) |

**Supplementary Table 6.**

| Generation of human pancreas organoid cultures and manipulation of cell polarity | | |
| --- | --- | --- |
| Component | Manufacturer | Cat.No. |
| Matrigel | Corning | 354234 |
| 24-well plate | Greiner | 662160 |
| TrypLE™ Express Enzyme | Gibco | 12605028 |
| Anti-Adherence Rinsing Solution | StemCell Technologies | 07010 |

**Supplementary Table 7.**

| Cryopreservation of primary epithelial cells | | |
| --- | --- | --- |
| Component | Manufacturer | Cat.No. |
| DMSO | Sigma | D5879-1L |
| 2-Propanol | Molar Chemicals | 00390 |
| Mr. Frosty | Sigma | C1562 |

**Supplementary Table 8.**

| Gene expression analysis by qRT-PCR | | |
| --- | --- | --- |
| Component | Manufacturer | Cat.No. |
| NucleoZOL | Macherey-Nagel | 740404 |
| NanoDrop One/OneC UV-Vis Spectrophotometer | Thermo Scientific | ND-ONEC-W |
| iScript cDNA Synthesis kit | Bio-Rad | 1708890 |
| SsoAdvanced Universal SYBR Green Supermix | Bio-Rad | 1725274 |

**Supplementary Table 9.**

| Gene expression analysis by qRT-PCR (primers) | | |
| --- | --- | --- |
| Gene | Fwd (5’-3’) | Rev (5’-3’). |
| *CFTR* | CTGGAGCAGGCAAGACTTCA | TTGGCATGCTTTGATGACGC |
| *ANO1* | TCACCAAGATCGAGGTCCCA | GCCACGTAAAAGATGGGGGT |
| *SCNN1A* | CCTGCAACCAGGCGAATTAC | ACCGTTGTTGATTCCAGGCA |
| *SCNN1D* | TGGGGTTCAGACTGTGCAA | CAGTCCAGGCCATCGTAACT |
| *B2M* | TGGAGGCTATCCAGCGTACT | CTCTGCTGGATGACGTGAGT |

**Supplementary Table 10.**

| Gene expression analysis of hPOCs by RNA-Seq | | |
| --- | --- | --- |
| Component | Manufacturer | Cat.No. |
| NucleoSpin RNA Plus kit | Macherey-Nagel | 740984.250 |

**Supplementary Table 11.**

| siRNA transfection | | |
| --- | --- | --- |
| Component | Manufacturer | Cat.No. |
| siGLO Green transfection indicator | Horizon | D-001630-01 |
| Lipofectamine 2000 | Invitrogen | 11668019 |
| Opti-MEM | Gibco | 31985070 |
| CFTR siRNA pool | Dharmacon | L-006425-00-0005 |
| ANO1 siRNA poo | Dharmacon | L-027200-00-0005 |
| SCNN1A siRNA pool | Dharmacon | L-006504-00-0005 |
| SCNN1D siRNA pool | Dharmacon | L-006506-00-0005 |

**Supplementary Table 12.**

| Immunofluorescent labeling for confocal microscopy | | |
| --- | --- | --- |
| Component | Manufacturer | Cat.No. |
| Shandon Cryomatrix | ThermoFisher S. | 6769006 |
| Cryostat | Leica | CM 1860 UV |
| Microscope slides | ThermoFisher S. | J3800AMNZ |
| PFA | Alfa Aesar | 43368 |
| PBS | Sigma | P4417-100TAB |
| Sodium Citrate | Sigma | 71402 |
| Tween-20 | Sigma | P1379 |
| Goat Serum | Sigma | G9023 |
| Bovine Serum Albumin (BSA) | Pan-Biotech | P061391100 |
| Anti-CFTR antibody | Abcam | ab2784 |
| Cytokeratin 19 Antibody | ThermoFisher S. | MA5-31977 |
| Anti-SLC4A4/NBC | Abcam | ab187511 |
| Occludin Monoclonal Antibody | ThermoFisher S. | 33-1500 |
| Recombinant Anti-SOX9 antibody | Abcam | ab185966 |
| Phalloidin | Abcam | ab176759 |
| Anti-HNF-1B antibody | Abcam | ab236759 |
| Recombinant Anti-FOXA2 antibody | Abcam | ab108422 |
| DOG-1 antibody | ThermoFisher S | MA5-16358 |
| alpha-ENaC antibody | ThermoFisher S | PA1-920A |
| Extracellular Anti-Orai1 antibody | Alomone Labs | ACC-062 |
| Anti-PIEZO1 antibody | Alomone Labs | APC-087 |
| Goat anti-Mouse Alexa 488 | ThermoFisher S. | A11001 |
| Goat anti-Rabbit Alexa 488 | ThermoFisher S. | A11034 |
| Donkey anti-Mouse Alexa 647 | ThermoFisher S. | A31571 |

**Supplementary Table 13.**

| Scanning Electron Microscopy (SEM) | | |
| --- | --- | --- |
| Component | Manufacturer | Cat.No. |
| Glutaraldehyde | Electron Microscopy Sciences | 16220 |
| Sodium Cacodylate Tryhidrate | Electron Microscopy Sciences | 12310 |
| Poly-L-Lysine | Merck Millipore | A-005-C |
| HMDS | Sigma | 440191 |

**Supplementary Table 14.**

| Fluorescent microscopy and reverse swelling assay | | | | | | |
| --- | --- | --- | --- | --- | --- | --- |
|  | | Applied solutions | | | | |
| Component | Manufacturee Cat.No. | Standard HEPES pH=7.5 | Ca^2+^ free HEPES pH=7.5 | Standard HCO_3_^-^ | Cl^-^ free HCO_3_^-^ | Na^+^ free HEPES  pH=7.5 |
| NaCl | Sigma, S9888 | 140 mM | 142 mM | 115 mM | - | - |
| KCl | Sigma, P3911 | 5 mM | 5 mM | 5 mM | - | 5 mM |
| MgCl_2_ | Sigma, M2670 | 1 mM | 1 mM | 1 mM | - | 1 mM |
| CaCl_2_ | Sigma, 223506 | 1 mM | - | 1 mM | - | 1 mM |
| HEPES | Sigma, H3375 | 10 mM | 10 mM | - | - | 10 mM |
| Glucose | Sigma, G8270 | 10 mM | 10 mM | 10 mM | 10 mM | 10 mM |
| NaHCO_3_^-^ | Sigma, S6014 | - | - | 25 mM | 25 mM |  |
| EGTA | Sigma, E4378 | - | 1 mM | - | - |  |
| Na-gluconate | Sigma, S2054 | - | - | - | 115 mM |  |
| K_2_SO_4_ | Sigma, P9458 | - | - | - | 2.5 mM |  |
| Mg-gluconate | Sigma, M7554 | - | - | - | 1 mM |  |
| Ca-gluconate | Sigma, C8231 | - | - | - | 6 mM |  |
| N-Methyl-D-glucamine | Sigma, M2004-500G | - | - | - | - | 140 mM |

**Supplementary Table 15.**

| Fluorescent microscopy and reverse swelling assay | | |
| --- | --- | --- |
| Component | Manufacturer | Cat.No. |
| Poly-L-lysine | Sigma | P8920-100ML |
| Cover glass | VWR | ECN 631-1583 |
| Fura2-AM | Invitrogen | F1201 |
| MQAE | Invitrogen | E3101 |
| BCECF, AM | Biotium | 51012 |
| SBFI, AM | Invitrogen | S1263 |
| CPA | Tocris | 1235 |
| GSK7975A | Sigma | 5.34351 |
| Forskolin | Tocris | 1099 |
| CFTRinh-172 | Biotechne | 3430 |
| VX-770 | Merck Millipore | 530541 |

**Supplementary Table 16.**

| Gene abbreviation | Gene name |
| --- | --- |
| *LGR5* | Leucine-rich repeat-containing G-protein coupled receptor 5 |
| *CFTR* | Cystic fibrosis transmembrane conductance regulator |
| *KRT19* | Cytokeratin 19 |
| *OCLN* | Occludin |
| *SOX9* | SRY-Box transcription factor 9 |
| *EPCAM* | epithelial cell adhesion molecule |
| *CDH1* | E-cadherin |
| *HES1* | Hes family BHLH Transcription Factor 1 |
| *AMY1A* | Amylase alpha 1A |
| *AMY1B* | Amylase alpha 1B |
| *AMY1C* | Amylase alpha 1C |
| *PPY* | Pancreatic polypeptide |
| *INS* | Insulin |
| *CHGA* | Chromogranin A |
| *CHGB* | Chromogranin B |
| *CDH5* | Vascular endothelial cadherin |
| VIL1 | Villin |
| PLS1 | Plastin1 |
| ESP | Espin1 |
| ACTG1 | Actin gamma 1 |
| MYO1A | Myosin 1A |
| MYO6 | Myosin 6 |
| EZR | Ezrin |
| SLK | STE20 like kinase |
| MYO7B | Myosin 7 B |
| USH1C | Harmonin |
| PIEZO1 | Piezo-Type Mechanosensitive Ion Channel Component 1 |
| CCNA1 | Cyclin A1 |
| CCNA2 | Cyclin A2 |
| CCNB1 | Cyclin B1 |
| CCNB2 | Cyclin B2 |
| CCNB3 | Cyclin B3 |
| CCND1 | Cyclin D1 |
| CCND2 | Cyclin D2 |
| CCND3 | Cyclin D3 |
| CCNE1 | Cyclin E1 |
| CCNE2 | Cyclin E2 |
| CCNQ | Cyclin M |
| ANO1-10 | Anoctamin 1-10 |
| SCNN1A | Sodium channel epithelial 1 subunit alpha |
| SCNN1B | Sodium channel epithelial 1 subunit beta |
| SCNN1D | Sodium channel epithelial 1 subunit delta |
| SCNN1G | Sodium channel epithelial 1 subunit gamma |
| ATP2B1 | ATPase Plasma Membrane Ca^2+^ Transporting 1 |
| ATP2B2 | ATPase Plasma Membrane Ca^2+^ Transporting 2 |
| ATP2B3 | ATPase Plasma Membrane Ca^2+^ Transporting 3 |
| ATP2B4 | ATPase Plasma Membrane Ca^2+^ Transporting 4 |
| ATP2A1 | ATPase Sarcoplasmic/Endoplasmic Reticulum Ca^2+^ Transporting 1 |
| ATP2A2 | ATPase Sarcoplasmic/Endoplasmic Reticulum Ca^2+^ Transporting 2 |
| ATP2A3 | ATPase Sarcoplasmic/Endoplasmic Reticulum Ca^2+^ Transporting 3 |
| STIM1 | Stromal Interaction Molecule 1 |
| STIM2 | Stromal Interaction Molecule 2 |
| ORAI1 | ORAI Ca^2+^ Release-Activated Calcium Modulator 1 |
| ORAI2 | ORAI Ca^2+^ Release-Activated Calcium Modulator 2 |
| ORAI3 | ORAI Ca^2+^ Release-Activated Calcium Modulator 3 |
| CAMK2A | Ca^2+^/Calmodulin dependent protein kinase II alpha |
| CAMK2B | Ca^2+^/Calmodulin dependent protein kinase II beta |
| CAMK2D | Ca^2+^/Calmodulin dependent protein kinase II delta |
| CAMK2G | Ca^2+^/Calmodulin dependent protein kinase II gamma |


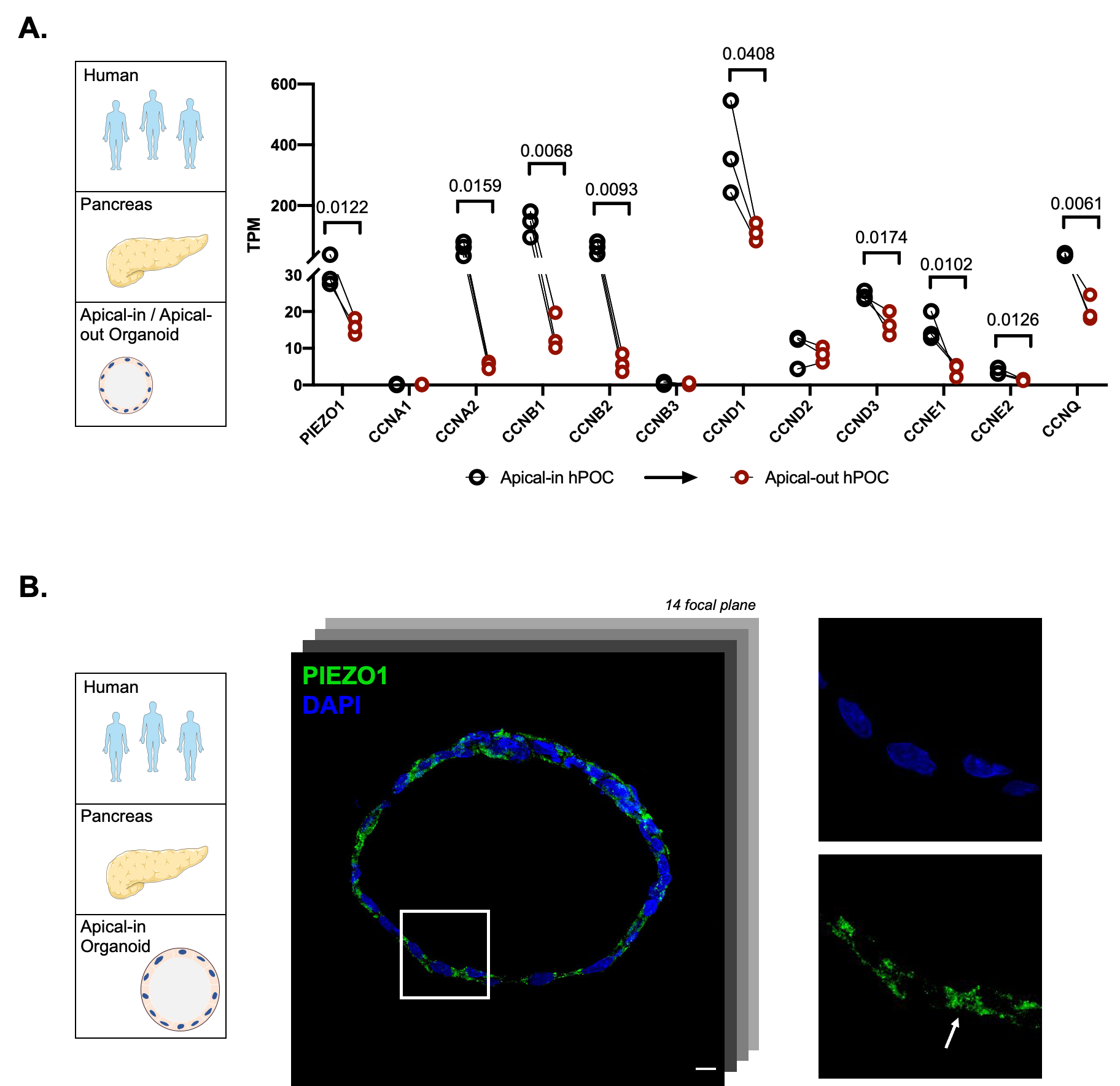


**Supplementary Figure 1. A.** Comparative transcriptome analysis of apical-in (N=3) and apical-out (N=3) human pancreatic organoid cultures by RNA sequencing. Data are indicated in TPM value (Transcript/million). Gene abbreviations are in **Supplementary Table 16**. **B.** Merged confocal (Z-stack) picture demonstrates the presence of PIEZO1 protein on Apical-in hPOCs. The highlighted detail and the arrow show the localization of PIEZO1 mainly between the elongated cells (scale bar: 10 µm).


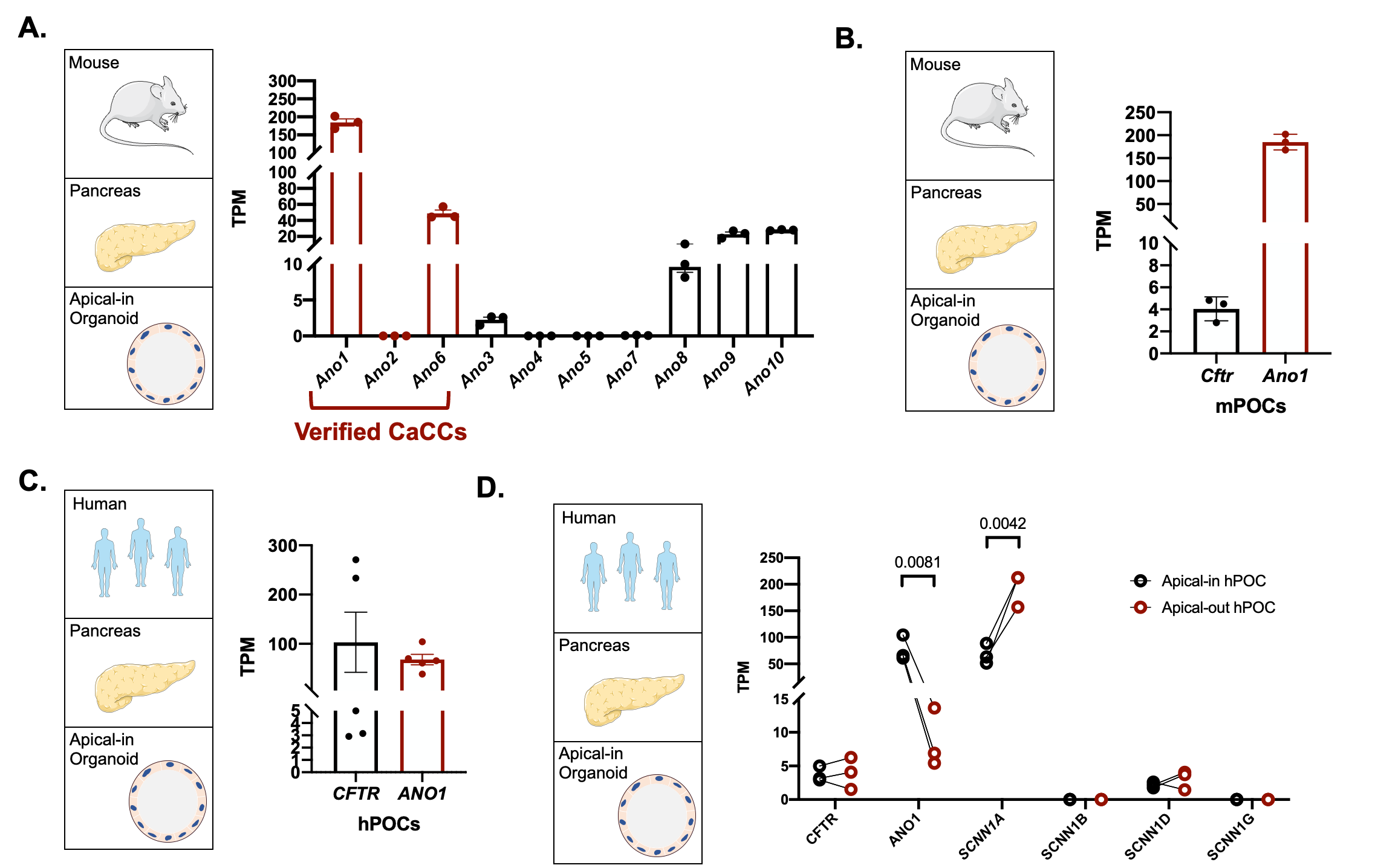


**Supplementary Figure 2. A.** Bar charts demonstrate RNA-seq profile of ANO family members in mouse (N=3) pancreas derived organoid cultures (mPOCs). Gene expression profile of *CFTR and ANO1* determined in mouse (**B.**) (N=3) and human (**C.**) (N=5) pancreatic organoids. D. Transcriptomic comparison of CFTR, ANO1, SCNN1A/B/D/G in in apical-in and polarity switched apical-out hPOCs (N=3). All data are indicated in TPM values (Transcript/million). Gene abbreviations are placed in **Supplementary Table 16**.

**Supplementary Figure 3.** Transfection control (siGLO Green) assay of apical-in organoids growing in ECM (Matrigel) and apical-out organoids maintained in suspension. The white arrow in the brightfield image marks the border of the Matrigel, while the white circles show the rare transfected cells that were able to take up siGLO Green indicator. Apical out organoids kept in suspension were perfectly able to take up the transfection indicator.

**Supplementary Figure 4.** Validation of the 48-hour-long gene silencing method carried out on apical-out organoids. The efficiency of the pre-validated siRNA pools on the target genes’ expression was verified at the level of mRNA by qRT-PCR (**A.**) and also at protein level by immunofluorescent labelling (**B.**). All qRT-PCR raw data was analyzed by the ΔΔCq method, while in case of IF confocal pictures, data for three individual intensity profiles were collected from three distinct membrane areas and maximum intensity values of the independent intensity profiles are plotted on bar charts (scale bar: 10 µm).
